# Breathable Textile Rectangular Ring Microstrip Patch Antenna at 2.45 GHz for Wearable Applications

**DOI:** 10.3390/s21051635

**Published:** 2021-02-26

**Authors:** Abdul Wahab Memon, Igor Lima de Paula, Benny Malengier, Simona Vasile, Patrick Van Torre, Lieva Van Langenhove

**Affiliations:** 1 Centre for Textile Science and Engineering, Department of Materials, Textiles and Chemical Engineering, Ghent University, 9052 Ghent, Belgium; Benny.Malengier@UGent.be (B.M.); Lieva.VanLangenhove@UGent.be (L.V.L.); 2 Department of Textile, Mehran University of Engineering & Technology, 76020 Jamshoro, Pakistan; 3 Department of Information Technology, Faculty of Engineering and Architecture imec-IDLab, Ghent University, 9052 Ghent, Belgium; Igor.LimadePaula@UGent.be (I.L.d.P.); Patrick.VanTorre@UGent.be (P.V.T.); 4 Fashion and Textiles Innovation Lab FTILab+, HOGENT University of Applied Sciences and Arts, 9051 Ghent, Belgium; Simona.Vasile@hogent.be

**Keywords:** microstrip patch antenna, wearable textile antenna, wearable wireless communication, breathable antenna, air permeability, water vapor permeability, ISM band

## Abstract

A textile patch antenna is an attractive package for wearable applications as it offers flexibility, less weight, easy integration into the garment and better comfort to the wearer. When it comes to wearability, above all, comfort comes ahead of the rest of the properties. The air permeability and the water vapor permeability of textiles are linked to the thermophysiological comfort of the wearer as they help to improve the breathability of textiles. This paper includes the construction of a breathable textile rectangular ring microstrip patch antenna with improved water vapor permeability. A selection of high air permeable conductive fabrics and 3-dimensional knitted spacer dielectric substrates was made to ensure better water vapor permeability of the breathable textile rectangular ring microstrip patch antenna. To further improve the water vapor permeability of the breathable textile rectangular ring microstrip patch antenna, a novel approach of inserting a large number of small-sized holes of 1 mm diameter in the conductive layers (the patch and the ground plane) of the antenna was adopted. Besides this, the insertion of a large number of small-sized holes improved the flexibility of the rectangular ring microstrip patch antenna. The result was a breathable perforated (with small-sized holes) textile rectangular ring microstrip patch antenna with the water vapor permeability as high as 5296.70 g/m^2^ per day, an air permeability as high as 510 mm/s, and with radiation gains being 4.2 dBi and 5.4 dBi in the E-plane and H-plane, respectively. The antenna was designed to resonate for the Industrial, Scientific and Medical band at a specific 2.45 GHz frequency.

## 1. Introduction

Textile architectures with embedded antennas have turned out to be an integral part of wearable device systems, enabling body-centric wireless communication with garments [1]. Wearable device systems can be (others are possible as well) monitoring systems dedicated to assisted living and lifecare. If these monitoring systems are integrated into textile clothing, they become wearable textile systems [2]. An antenna is a part of a monitoring system responsible for wireless communication [3]. Athletes, mountaineers, miners, military, rescuers, firefighters, and many other outdoor users need healthcare and navigation information to be transferred wirelessly to a base station to monitor their health conditions. In hospitals, patients whose life is at risk need to be monitored within safe zones every time. Body-centric devices attached to the garment of patients can notify doctors and nursing personnel wirelessly about their health condition, which helps them to look after their patients more effectively and take immediate action if needed. Effective information transmission can be achieved through antennas embedded into garments, termed wearable antennas [3,4]. Textile patch antennas, among a wide range of different types of antennas, are the most popular antenna topology for wearable applications. Their planar structure, simple design, flexibility, low weight, and ease of integration into any garment make these antennas suitable for wearable applications [5]. A Microstrip Patch Antenna (MPA) design includes a non-conductive textile substrate, also called the dielectric substrate, sandwiched between the conductive patch and the conductive ground plane. Furthermore, the presence of a conductive ground plane layer negates any adverse effect on the human skin due to the back radiation of an antenna [5,6].

Physical factors such as bending [7], stretching [8,9,10,11] and temperature [12,13] tend to shift the resonance frequency of an antenna. Because of the hydrophilic character of textiles, textile antennas are affected more by humidity compared to non-textile-based antennas such as FR4, RT/Duroid and hydrophobic foam. Textile materials used as an antenna substrate may transport humidity between the body and the atmosphere and absorb part of it. Water has a high relative permittivity (Ɛ_r_ = 78) and, in comparison, the relative permittivity of dry textile substrates ranges between 1 and 2 [14]. When the humidity is absorbed by the textile substrate, the relative permittivity of the textile substrate increases due to the high permittivity of water together with its high conductivity, which results in the shift of the resonance frequency of the textile antenna towards the lower side [15,16]. The amount of absorbed humidity depends upon atmospheric temperature, moisture content and moisture regain of the textile material used to construct the textile antenna. To combat the adverse effect of humidity on the performance of a textile antenna during its wearability, the selected textile substrate should be hydrophobic or has the least moisture uptake ability and should have the ability to transport the humidity effectively to the environment. However, the hydrophobic nature of the textile substrate may cause an uncomfortable feeling to the wearer because no humidity will be absorbed by the textile substrate and the humidity will remain trapped underneath the antenna. Therefore, the high water vapor permeability (WVP) would be a solution to prevent the accumulation of humidity underneath and inside the textile antenna.

A breathable textile antenna can be an essential substitute to conventional textile antennas and can cope with the above-mentioned working strains more effectively. The breathability is a factor of porosity, the higher the porosity of materials used to construct an antenna, the more an antenna becomes breathable [17]. The high air permeability (AP) of textiles can effectively dissipate humidity back into the environment [18,19]. A breathable textile antenna can also be effective in providing ease to the wearer at a time when the wearer performs any physical activity. In [20], the porous patch antenna is constructed through screen printing of conductive silver ink on a highly absorbent engineered Evolon^®^ textile substrate to help the silver ink to penetrate evenly over its surface through a strong capillary wicking force. The antenna, later on, is packaged with a porous polyurethane web as an additional process to make the antenna durable, breathable, and water repellent, which is an additional, delicate and expensive step to avoid the effect of humidity over the textile antenna. Previous literature on wearable textile antennas so far has been limited to a number of discrete properties like dual-band antenna [21,22,23,24], bending effect on the resonance frequency of the antenna [25,26], usage of novel conductive electrotextile fabrics to construct textile patch antennas [27], and comparison of different textile properties [28]. Another important aspect is the physical performance of the wearer of a textile antenna, which can be affected if a textile antenna, despite its relatively small size, resists the transportation of humidity and/or perspiration and remain trapped inside a textile antenna can be uncomfortable even to a little extent for the wearer. This is particularly the case when high-intensity activities are performed with large sweat production as a consequence. The perspiration also affects the electrical properties and the dimensional stability of the textiles [12,15]. The height of the textile substrate changes as the perspiration swells the textile substrate. If the change in the height of the textile substrate occurs, the antenna will no more resonate at the desired frequency. Some textile fabrics shrink more and some less when they make contact with water, which means they change their physical dimensions when exposed to water. Therefore, a textile substrate with low values of moisture regain and moisture content may overcome the change of dimensional stability issue.

**Figure 1:**
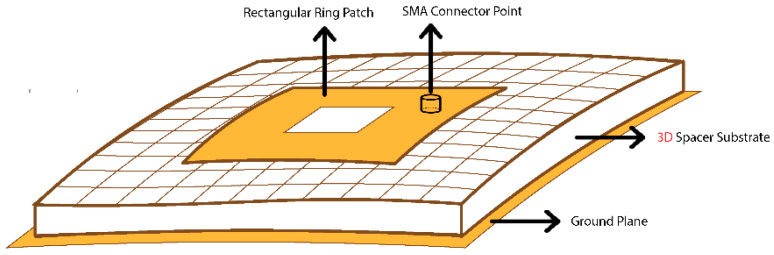
Microstrip rectangular ring patch antenna.

A microstrip patch antenna (MPA) has a relatively smaller bandwidth, hence some special techniques have been followed to increase the bandwidth of the antenna. One technique is to cut a rectangular patch from the center of the MPA [29,30], which results in a rectangular ring microstrip patch antenna (RRMPA). In this study, the RRMPA topology has been selected as illustrated in Figure 1. This research demonstrates a novel approach of constructing a breathable perforated textile antenna through added perforations in the conducting elements of RRMPA, which improves its WVP so that the humidity under and inside the antenna substrate can be effectively transported back to the environment. To validate the approach, this paper follows a number of basic steps to develop a breathable textile antenna, employing a highly perforated 3-dimensional (3D) knitted spacer fabric as the textile substrate (perforations are added during the knitting process) and the conductive layers (the ground plane and the patch) with regular perforations/holes (added through laser cutting during the construction of the textile antenna). All the antenna prototypes are designed to resonate at the Industrial, Scientific, and Medical (ISM) frequency band ranges from 2.4 GHz to 2.4835 GHz, which covers 83.5 MHz of bandwidth. It should be noted that an extra benefit of an RRPMA is that the ring structure creates a hole within the antenna patch, allowing more air and water vapors to pass through the antenna, which helps to enhance the breathability of the antenna.

## 2. Materials and Methods

### 2.1. D Dielectric Substrate

**Figure 2:**
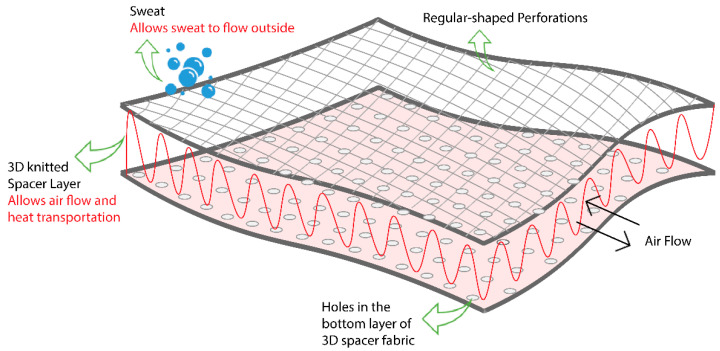
Structural diagram of the 3D knitted spacer substrate.

The selected 3D knitted spacer antenna substrate is made of polyester and nylon that have very low moisture regain and moisture content values of 0.54% and 0.42% [31], respectively. Furthermore, the substrate is flexible and dimensionally stable enough to withstand rough handling and other stresses like bending and stretching during construction and harsh use of the antenna. The structural view of the 3D substrate is illustrated in Figure 2. The open mesh structure or the regular perforations in the 3D spacer substrate creates plenty of air cavities within the substrate, allowing easy passage for air and water vapors to pass through that helps to make the textile antenna breathable.

The high void ratio of a regularly perforated 3D substrate in terms of porosity ensures very low dielectric losses, as most of the volume within the substrate is occupied by air. The ratio of the total volume of voids to the entire volume of solid including voids is termed as the void ratio of that solid [17,32]. A large number of voids or air cavities brings the dielectric constant of the perforated substrate closer to the dielectric constant of air and also results in lower dielectric losses [33].

### 2.2. Conductive Materials

Flectron^®^ is a conductive copper-coated nylon fabric and other conductive tapes constituting different conductive materials like copper, nickel, and silver that has been used in previous research on textile antennas [34,35,36]. In this study, three different conductive fabrics from the Shieldex brand (produced by Statex Produktions, Germany) have been used as the conductive patch and conductive ground plane for the antenna construction. Shieldex^®^ Prag, Shieldex^®^ Kiel-SK-96, and Shieldex^®^ Koln are copper-coated nylon fabrics with a corrosion-resistant coating. They have different fabric structures, AP values, weight, and surface resistivity values.

**Figure 3:**
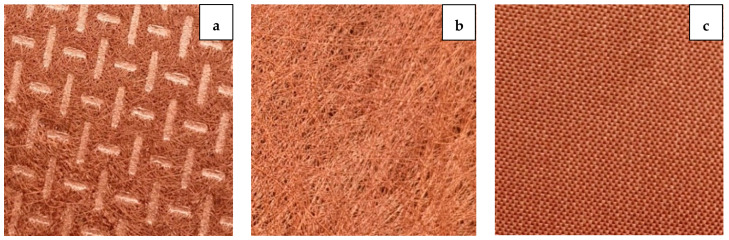
Cloth construction of (**a**) Shieldex^®^ Kiel-SK-96, (**b**) Shieldex^®^ Koln, and (**c**) Shieldex^®^ Prag conductive fabrics.

The properties of Shieldex^®^ conductive metalized fabrics collected from data sheets provided by Statex Produktions are summarized in Table 1. The selected conductive fabrics have very low surface resistivity and have different fabric constructions to search for the most suitable geometry to construct a textile breathable RRMPA. Shieldex^®^ Prag is a compact structured woven conductive fabric with almost no visible gaps between yarns with the lowest AP among the other two conductive fabrics. Shieldex^®^ Koln and Shieldex^®^ Kiel-SK-96 are nonwoven fabrics that have higher air permeabilities compared to Shieldex^®^ Prag. Shieldex^®^ Prag is a, Figure 3 illustrates the cloth construction of all the three conductive fabrics by Shieldex^®^ used in this research work.

To further enhance the breathability of the antenna, several holes of 1 mm diameter size are inserted with equal spacing in both the conductive patch and the ground plane of the RRMPA. Insertion of a large number of small-sized holes in conductive layers of the antenna increases direct air passages, making the antenna more air-permeable, and therefore will help to get more WVP. Additionally, the insertion of holes in the conductive layers enhances the overall mechanical flexibility of the antenna which helps the wearer feel more comfortable [14]. The integration of a large number of small-sized holes in conductive layers of the antenna in combination with a high perforated textile substrate and adaptation of the RRMPA topology should make the antenna breathable, mechanically flexible, robust, and suitable for wearable applications. This particular approach of making a textile breathable antenna on this particular antenna design is novel.

### 2.3. Characterization of the Dielectric Substrate

The 3D perforated knitted spacer substrate constitutes polyester and nylon. The thickness of the 3D perforated textile substrate is determined through the ISO 5084 standard. Thickness measurements were performed with a Digimatic Indicator from Mitutoyo which has a circular pressure plate of 3 mm diameter that applies 20 kPa pressure.

The dielectric constant (Ɛ_r_) and loss tangent (tan δ) of the 3D textile substrate are measured through the resonance method [37]. An iterative process based on the resonance frequency (resonance method) was followed to characterize the 3D textile dielectric substrate at the 2.45 GHz operating frequency using the CST studio suite, an electromagnetic and multiphysics simulation software. To characterize the 3D substrate, at the start, an arbitrary value of Ɛ_r_ is selected only to design and simulate the antenna as the real value of Ɛ_r_ for the 3D substrate was unknown and needed to be characterized. Accordingly, the antenna was simulated for the 3D substrate at 2.45 GHz operating frequency. Later on, the antenna was constructed and measured for its reflection coefficient through Vector Network Analyzer (VNA) and the results were compared with its simulated reflection coefficient. Initially, there appeared a difference between resonance frequencies of both the simulated and measured reflection coefficients of the antenna. Next, the chosen arbitrary value to the Ɛ_r_ during the simulation was adjusted until the resonance frequency of the simulated antenna coincides with the resonance frequency of the constructed antenna. Once the resonance frequency of the simulated antenna at the new value of Ɛ_r_ coincides with the resonance frequency of the constructed antenna, that new value of Ɛ_r_ is considered as the actual value for the 3D substrate. The presence of substrate losses results in a broader and less deep resonance peak in the return loss graph [1]. The antenna was simulated at a value of 0 for tan δ. The antenna prototype was manufactured and return loss characteristics were measured employing a VNA. The depths of resonance peaks for both simulated and measured return loss characteristics were compared. The value of the tangent loss was varied until both peaks coincide. Measured characteristics for the 3D perforated textile substrate at 2.45 GHz frequency are mentioned in Table 2.

According to [38], regular perforations reduce substrate losses, thereby improving antenna performance. The 3D knitted spacer substrate also offers better flexibility and elastic behavior as the nylon content offers excellent elastic properties.

### 2.4. Water Vapor Transmission Rate of Antennas

The water vapor transmission behavior of textiles is always of importance when it comes to thermophysiological comfort [39]. The water vapor transmission rate (WVTR) test evaluates the transfer of water vapors through semi-permeable or permeable samples. The WVTR is the steady flow of water vapor in unit time through a specific area under specified temperature and humidity conditions and is expressed in grams per square meter per day (g/m^2^ × day). The WVTR of breathable antennas is measured through the ISO 2528:2017 gravimetric method. A dish of 50 cm^2^ area with a desiccant in the form of granules (anhydrous calcium chloride—CaCl_2_) is used to measure WVTR enclosed with a test specimen (an antenna) placed in a controlled atmosphere (38 °C ± 1° and 90% ± 2% relative humidity). For each antenna type, three circular test specimens were tested and the mean WVTR value was calculated. The WVTR test validates the purpose of breathable textile antennas if they can transmit water vapors around them back to the atmosphere while used practically. The WVTR test result for each antenna is calculated through

$$WVTR\;\left(\frac{g}{m^{2}\times day}\right)=\;\frac{24\;\times 10^{4}\;\times m₂}{s\;\times t}$$

where *t* is the total duration, in hours, of the last two stable exposure readings, *m_₂_* is the increase in mass, in grams, of the assembly during the time *t*, *s* is the area of the tested surface (normally 50 cm^2^).

### 2.5. Air Permeability of Antennas

The AP of the dielectric substrate and the conductive fabrics employed in constructing antenna prototypes is measured through the ISO 9237:1995 standard. This method measures the permeability of fabrics to air and applies to most types of fabrics, including industrial fabrics. This method uses a 100 Pa pressure differential for apparel and a 200 Pa pressure differential for industrial fabrics at the relative humidity of 50% and a temperature of 23 °C. The AP of antennas was measured at a 200 Pa pressure differential on the Permeabilimeter with a tested surface area of 20 cm^2^. The AP outcome of the dielectric substrate is tabulated in Table 1, whereas the AP outcomes of individual conductive fabrics are tabulated in Table 2, respectively. 

## 3. Antenna Design and Simulation

In this section, the CST Studio Suite, an Electromagnetic and Multiphysics simulation software, is used to design and simulate the RRPMA with its high perforated 3D spacer substrate. CST Studio Suite offers a wide range of tools for designing, analyzing and optimizing antennas.

### 3.1. Antenna Modeling and Simulation

**Figure 4:**
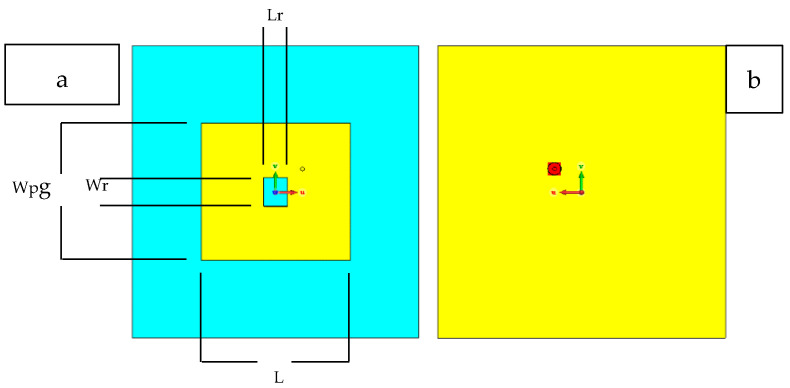
Front view (**a**) and back view (**b**) of the CST model of the non-perforated RRMPA.

The resonance frequency of the RRMPA is chosen at 2.45 GHz during the antenna design. The approximate dimensions of the antenna patch can be computed based on the resonance frequency, the dielectric constant of the substrate and the thickness of the substrate. The modeled 3D prototype of the RRMPA is then established in CST Studio. In this model, the coaxial feeding technique is followed in the construction of RRMPA. An SMA connector with 50 Ω characteristic impedance is used to feed power to the antenna and its location is at the diagonal of the antenna patch. Two different antenna models are simulated and later constructed to measure their performance. The first simulated model was the non-perforated RRMPA model obtained after optimization for its dimensions in the CST software and is shown in Figure 4.

**Figure 5:**
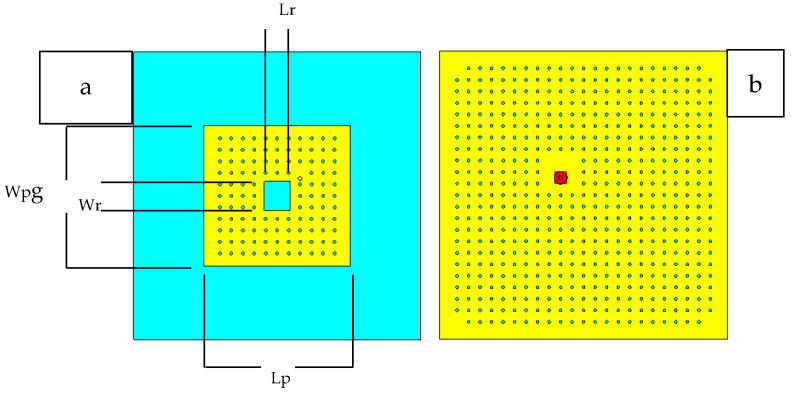
Front view (**a**) and back view (**b**) of the CST model of the perforated RRMPA.

**Figure 6:**
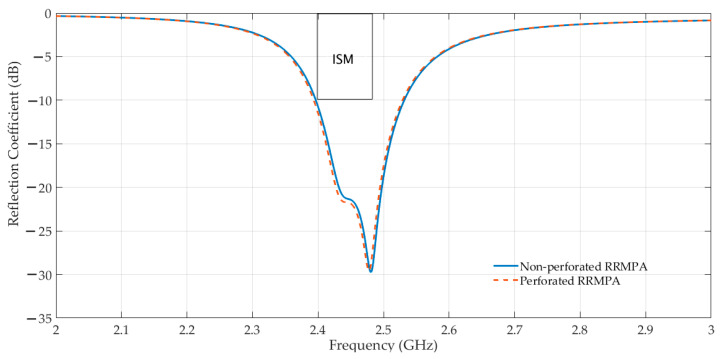
Simulated reflection coefficients (S11) of non-perforated and perforated RRMPAs.

The same procedure was followed to design the second simulated model, the perforated RRMPA. For this model, an additional step was present, namely a large number of small-sized holes (perforations) of 1 mm diameter were cut off the patch and the ground plane of non-perforated RRMPA. During the simulations of perforated RRMPA (with around 120 holes in the patch and around 500 holes in the ground plane of the perforated RRMPAs), it is observed that a negligible change in its simulated reflection coefficient curve is achieved compared to the simulated reflection coefficient curve of non-perforated RRMPA. This indicates that the addition of holes in the conductive layers of the non-perforated RRMPA during simulation does not change the reflection coefficient performance of the antennas. Therefore, no optimization of the dimensions for the patch of the perforated antenna is needed and the dimensions remain the same for both the patches of the non-perforated and perforated RRMPAs. The dimensions of the patch (non-perforated and perforated) are indicated in Table 3. Both the non-perforated and perforated simulated models of RRPMAs resonate at 2.45 GHz. The simulated perforated RRMPA model is shown in Figure 5. Furthermore, the contrast between the simulated reflection coefficients for the non-perforated and the perforated RRMPAs are illustrated in Figure 6 indicating a very slight difference in the curve of perforated RRMPA against the non-perforated curve.

The direction of electromagnetic radiations radiated by the antenna is represented by its radiation pattern. These patterns represent the distribution of the radiated energy by the antenna into space, as a function of direction. The radiation patterns of the simulated RRMPAs are taken from the CST Studio Suite software whereas the radiation patterns of the constructed antennas are measured in the anechoic chamber.

### 3.2. Construction of Antennas

The resonance frequency of the constructed RRMPAs is measured on Agilent E5071C Vector Network Analyzer (VNA). The power is transmitted to the antenna by VNA through a coaxial cable. The antenna reflects part of the power to the VNA due to impedance mismatching. The return loss, (S11), is the measure of the effectiveness of power delivery from a transmission medium to an antenna and is expressed in decibels (dB) [40]. Return loss attains minimum decibels at a specific resonance frequency of an antenna, which means the antenna utilizes most of the power to resonate at that specific frequency and the least power is reflected to the VNA. The return loss of −10 dB is the minimum value considered to be a good antenna, which indicates that 90% of the power is utilized by the antenna to resonate at the specific frequency (2.45 GHz is the specific frequency selected in this paper). 

**Figure 7:**
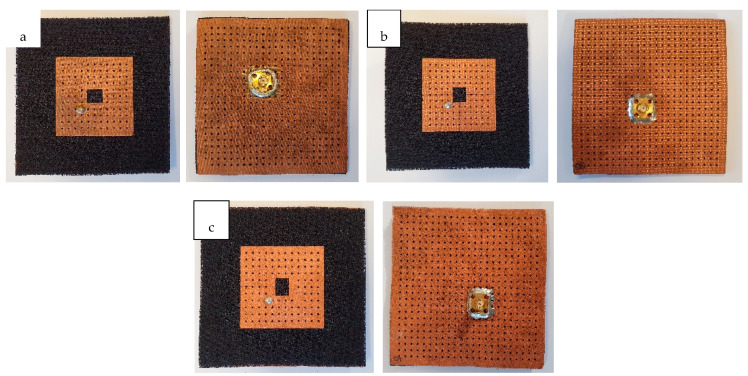
Front side (**left**) and backside antenna (**right**) (**a**) Prag-based perforated RRMPA, (**b**) Kiel-based perforated RRMPA, and (**c**) Koln-based perforated RRMPA.

Three non-perforated RRMPAs from all three Shieldex^®^ conductive fabrics and three perforated RRMPAs from the same Shieldex^®^ fabrics were constructed. All three conductive electro textiles, the 3D substrate and small holes in the patch and the ground plane for the perforated RRMPAs were precisely cut through laser cutting. All the non-perforated and the perforated RRMPAs were constructed by pasting dedicated conductive fabrics over the 3D dielectric spacer substrate with a porous adhesive layer through the heat press. Figure 7 illustrates the front and the back sides of all the constructed perforated RRMPAs employing (a) Shieldex^®^ Prag, (b) Shieldex^®^ Kiel-SK-96, and (c) Shieldex^®^ Koln, respectively.

All the constructed non-perforated and perforated RRMPAs cover the sufficient bandwidth required for the ISM band. The width and length of the substrate are similar to the width and length of the ground plane. Every hole inside the patch and the ground plane of the perforated antenna is of the same diameter. The effect of added perforations in the patch and the ground plane of all perforated RRMPAs on the WVP and the AP compared to the WVP and AP of non-perforated RRMPAs, and the effect on their reflection coefficients and gain patterns, as well as the comparison between the simulated and real measurements, are discussed in Section 4.

The antenna that covers the ISM band entirely with a minimum of 83.5 MHz of bandwidth from 2.40 GHz till at 2.4835 GHz frequency range is set as a minimum requirement in terms of the bandwidth. As will be given in Section 4, all the antennas constructed in this research work meet these criteria comfortably and achieve much higher bandwidth than the minimum set bandwidth requirement.

## 4. Results and Discussion

### 4.1. Reflection Coefficients (S11) of Antennas

**Figure 8:**
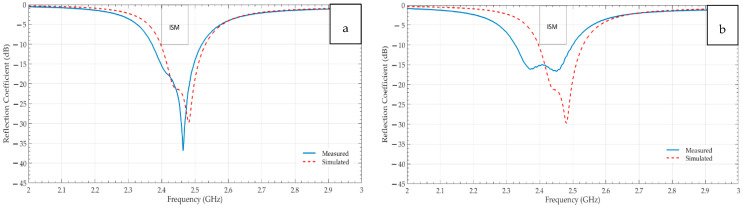
Simulated and measured reflection coefficients-S11 of (**a**) non-perforated PRAG based RRMPA (**b**) perforated PRAG based RRMPA.

The electromagnetic performance of the Shieldex^®^ Prag-based (a) non-perforated and (b) perforated RRMPAs in terms of their reflection coefficients is illustrated in Figure 8, respectively. Here, the comparison between the experimental and the simulated reflection coefficients is carried out to see how well both the antennas show resemblance in terms of the 2.45 GHz resonance frequency and the bandwidth. Graphs indicate that both constructed antenna types radiate at the required frequency of 2.45 GHz, attain a minimum of the −10 dB curve dip at that frequency effectively and sufficiently cover the entire ISM band, resulting in the bandwidths of 150 MHz and 180 MHz for the non-perforated and the perforated Shieldex^®^ Prag based RRMPAs, respectively. The requirement to achieve a minimum of 83.5 MHz bandwidth is achieved comfortably by both antennas. From the graphs, it can also be observed that the experimental return loss characteristics confirm better agreement (achieve a minimum of resonance frequency curve dip of −10 dB and cover entire ISM band) with simulated return loss characteristics of both non-perforated and perforated antennas. The reflection coefficient curve of the experimental perforated Shieldex^®^ Prag-based RRMPA varies from its simulated reflection coefficient curve. This may be because of the way of manufacturing the antennas (how well the SMA connector is soldered to the antenna, the soldering at proper feed location, rough handling and improper pasting of the conductive layers over the substrate) and limitations of the CST simulation software as the software does not take the perforations of the 3D perforated substrate into considerations. Besides all this, the experimental results of the perforated Shieldex^®^ Prag based RRMPA achieve the minimum set of requirements and is considered as a good antenna as it covers the entire ISM band and attains −10 dB return loss curve dip at 2.45 GHz frequency.

**Figure 9:**
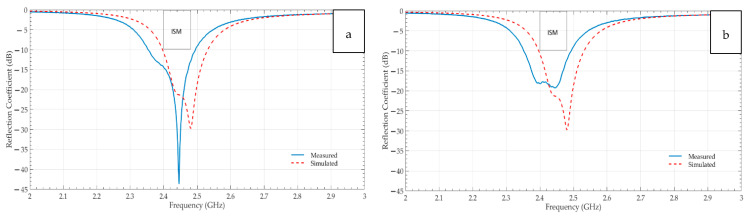
Simulated and measured reflection coefficients-S11 of (**a**) non-perforated KIEL based RRMPA (**b**) perforated KIEL based RRMPA.

Likewise, Figure 9 contrasts the experimental and the simulated return loss characteristics of (a) the non-perforated and (b) the perforated Shieldex^®^ Kiel-SK-96 based RRMPAs. The experimental results show better agreement with the simulated results in terms of the return loss characteristics for both the non-perforated and the perforated RRMPAs. It can be seen from the graphs that both the antennas attain a −10 dB return loss curve dip at 2.45 GHz frequency, effectively indicating that most of the power is utilized by the antennas to resonate giving high antenna performance. The bandwidths of 130 MHz and 140 MHz for the non-perforated and the perforated Shieldex^®^ Kiel-SK-96 based RRMPAs are achieved, respectively. Therefore, Shieldex^®^ Kiel-SK-96 based RRMPAs meet all the minimum set of requirements.

**Figure 10:**
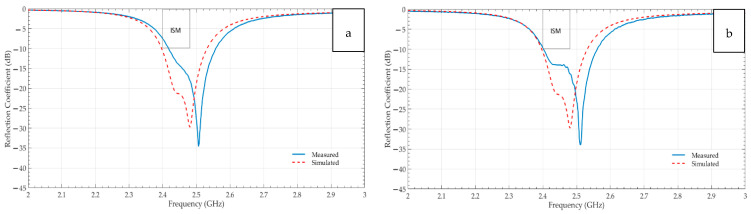
Simulated and measured reflection coefficients-S11 of (**a**) non-perforated KOLN based RRMPA (**b**) perforated KOLN based RRMPA.

At last, the graphical comparison between the experimental and the simulated return loss characteristics of the Shieldex^®^ Koln-based non-perforated and perforated RRMPAs is shown in Figure 10a,b, respectively. The frequency dip for the non-perforated RRMPA starts from 2.42 GHz and ends at 2.56 GHz. It implies that it resonates at the required 2.45 GHz resonance frequency by achieving the minimum −10 dB return loss at that frequency and offers a bandwidth of 140 MHz but its resonance dip starts slightly after the starting frequency of the ISM band. Whereas the perforated RRMPA covers the entire ISM band with a bandwidth of 160 MHz, it achieves the 2.45 GHz resonance frequency dip well below the −10 dB return loss range.

All constructed RRMPAs meet the basic criteria except the Shieldex^®^ Koln-based non-perforated RRMPA as it slightly misses to cover the entire ISM band but still, its 2.45 GHz resonance frequency is achieved.

### 4.2. Air Permeability of Antennas

AP of the antennas is determined by both the substrate and the conductive fabrics selected in this research work. The increasing AP values (low to high) of all three selected conductive fabrics from Shieldex^®^ were the defining factors for their selection to see the effect on the WVP of the antennas. To the non-perforated antennas, the combination of these three conductive fabrics having increasing AP values with the very different nature of the 3D knitted spacer substrate having regular perforations will justify the effect of increasing AP on their WVP. Furthermore, to the perforated antennas, the effect of the insertion of small-sized holes of 1 mm diameter in the conductive layers of antennas on their AP values is determined. The AP of RRMPAs is determined through the ISO 9237 standard to see how much an increase in the rate of airflow of perforated antennas is achieved after adding a large number of small-sized holes compared to the airflow rate of the non-perforated antennas. The outcomes of the AP results are summarized in Table 4 for all the non-perforated and perforated RRMPAs.

Among all three non-perforated antennas, the Koln-based RRMPA results in the highest AP of 400 ± 2% mm/s and the Prag-based RRMPA results in the least AP of 158 ± 2% mm/s. The increasing AP values of the three conductive fabrics justify that the structure of conductive fabrics also plays part in increasing the AP of non-perforated RRMPA without affecting their electromagnetic properties. Shieldex^®^ Koln-based non-perforated RRMPA offers more AP due to an open nonwoven structure. Whereas, the AP of the other two (Keil-based and Prag-based) non-perforated RRMPAs decreases as their structure becomes denser allowing less air to flow off them.

When the AP of the perforated RRMPAs was measured, a drastic change was observed for all three perforated RRMPAs. The highest change in percentage (more than 200%) was observed in the Prag-based perforated antenna compared to the non-perforated antenna of its kind. This is because when the small-sized holes are inserted in the compact woven structure of the Shieldex^®^ Prag, it drastically allows air to pass through it. Still, the overall AP of 322 ± 2% mm/s for the Prag-based RRMPA remains at the lower side compared to the other two perforated antennas. An AP of 510 ± 2% mm/s for the Koln-based perforated antenna was achieved, which is higher than the remaining two perforated RRMPAs. This is because the Shieldex^®^ Koln fabric has an advantage over the other two fabrics, having a higher starting AP than the other two conductive fabrics. It can be concluded from the AP results that the perforations remarkably improved the air-flow within the antennas without affecting much the antenna performance.

To validate these conclusions scientifically, the analysis of variance (ANOVA) on the AP test results of all the non-perforated and perforated antennas was performed at a 95% confidence interval which compares the joint effect of all the variables together statistically. For that, Minitab Statistical Software was used for the statistical analysis of the AP results. The AP values of the non-perforated and perforated RRMPAs are categorized as groups A and group B, respectively. The ANOVA is performed to check for any significant difference among the antennas in terms of their AP outcomes. The one-way ANOVA of all non-perforated antennas resulted in a significant difference with an F-value of 510.40 and a *p*-value of <0.001. Similarly, the difference is significant based on the one-way ANOVA of all perforated antennas with an *F*-value of 7885.79 and a *p*-value of <0.001. The change in *F*-value is a decisive factor between non-perforated and perforated RRMPAs as *F*-value tells about the significant difference between means of non-perforated and perforated AP values. Adding a large number of small size holes in conductive layers of perforated RRMPAs increases their AP dramatically, which is confirmed by the higher F-value of perforated antennas compared to the *F*-value of the non-perforated antennas. According to Tukey pairwise comparisons, there is a significant difference between all non-perforated and perforated antennas in terms of their AP results, which indicates the scientific validation of perforations in the perforated antennas over non-perforated antennas. The results of the analysis of variance for AP tests are tabulated in Table 5.

### 4.3. Radiation Patterns of Antennas

**Figure 11:**
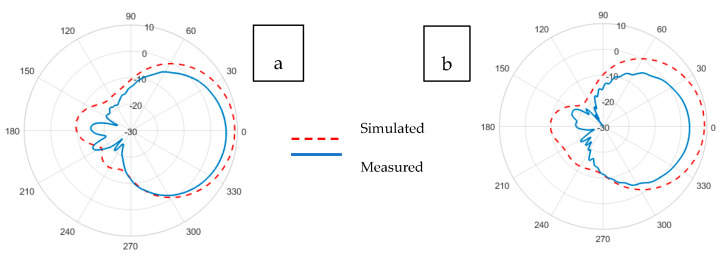
Simulated and experimental (**a**) E-plane and (**b**) H-plane radiation patterns of Prag-based non-perforated RRMPA at 2.45 GHz.

**Figure 12:**
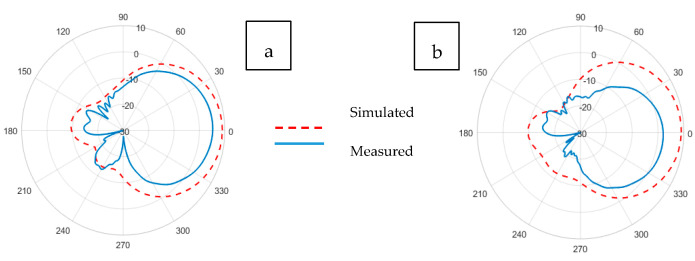
Simulated and experimental (**a**) E-plane and (**b**) H-plane radiation patterns of Prag-based perforated RRMPA at 2.45 GHz.

**Figure 13:**
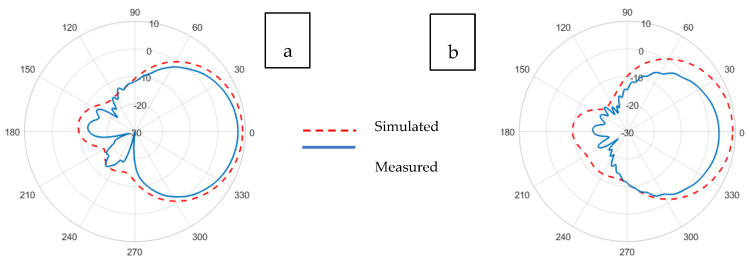
Simulated and experimental (**a**) E-plane and (**b**) H-plane radiation patterns of Kiel-SK-96-based non-perforated RRMPA at 2.45 GHz.

**Figure 14:**
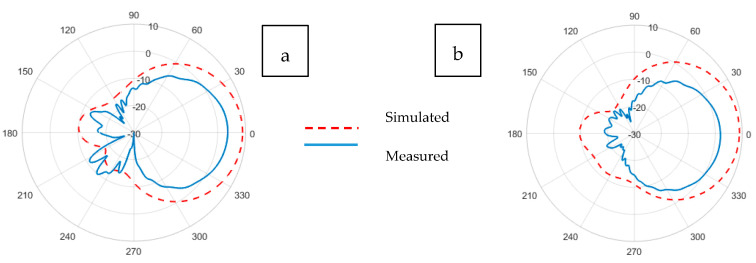
Simulated and experimental (**a**) E-plane and (**b**) H-plane radiation patterns of Kiel-SK-96-based perforated RRMPA at 2.45 GHz.

**Figure 15:**
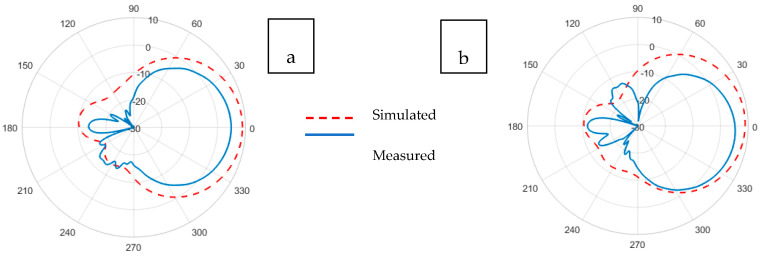
Simulated and experimental (**a**) E-plane and (**b**) H-plane radiation patterns of Koln-based non-perforated RRMPA at 2.45 GHz.

**Figure 16:**
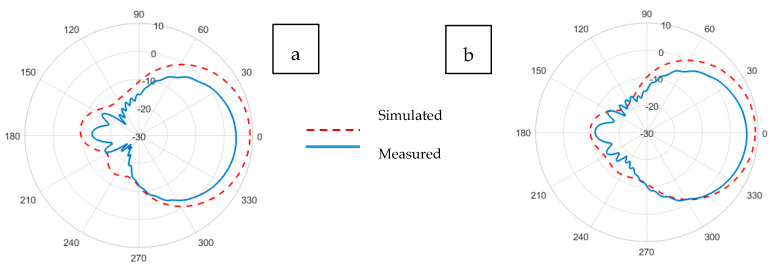
Simulated and experimental (**a**) E-plane and (**b**) H-plane radiation patterns of Koln-based perforated RRMPA at 2.45 GHz.

The radiation pattern of an antenna is the representation of the angular distribution of radiated power density by the antenna. The radiation pattern can simply be understood as a representation of the tendency of an antenna to radiate electromagnetic energy as a function of direction in the far-field region [41]. Whereas the antenna gain describes how much power is transmitted in the direction of peak radiation to that of an isotropic source and is expressed in dBi. The radiation patterns illustrated in Figure 11, Figure 12, Figure 13, Figure 14, Figure 15 and Figure 16 reflect the realized antenna gain that includes material and mismatch losses at both the E-plane (vertical) and the H-plane (horizontal), respectively. For a linearly polarized antenna, the plane having the electric-field vector and the direction of maximum radiation is termed E-plane, whereas the plane having a magnetic-field vector and the direction of maximum radiation is called H-plane [41].

Figure 11 and Figure 12 illustrate the realized gain patterns of the simulated and experimental (a) E-plane and (b) H-plane Prag-based non-perforated and perforated RRMPAs, respectively. For non-perforated Prag-based RRMPA, the gains of 5.8 and 3.3 dBi in the E-plane and H-plane, respectively, were obtained; whereas, for perforated Prag-based RRMPA, a gain of 5.3 dBi in the E-plane and 2.0 dBi in the H-plane were observed. It can be observed from both the illustrations, qualitatively, that the simulated gain patterns at both (a) E-planes and (b) H-plane are almost similar for both Prag-based non-perforated and perforated RRMPAs. As observed, a reduction in the realized gain of the Prag-based perforated RRMPA in both the E-plane and H-plane is seen compared to the non-perforated RRMPA of its kind. The reduction in the realized gain of Prag-based perforated is due to its higher back radiation. The back radiation in perforated RRMPA is because of the insertion of a large number of holes in the ground plane, as could be expected.

Illustrations for the realized gain patterns of the simulated and experimental Kiel-based non-perforated and perforated RRMPAs at (a) E-plane and (b) H-plane, respectively, are shown in Figure 13 and Figure 14. Gains of 7.3 in the E-plane and 4.0 dBi in the H-plane were observed for Kiel-based the non-perforated RRMPA; whereas, a gain of 3.6 dBi at the E-plane and 1.9 dBi at the H-plane were achieved for Kiel-based perforated antenna, respectively. However, due to the back radiation of the Kiel-based perforated RRMPA, a lower gain is obtained, compared to the gain of the Kiel-based non-perforated antenna of its kind.

The gain patterns for the simulated and experimental Koln-based non-perforated and perforated RRMPAs at (a) E-plane and (b) H-plane are shown in Figure 15 and Figure 16, respectively. Gains of 4.9 dBi and 5.9 dBi were achieved for Koln-based non-perforated RRMPA in the E-plane and H-plane, respectively; whereas, gains of 4.2 dBi in the E-plane and 5.4 dBi in the H-plane were observed for Koln-based perforated RRMPA. Once again, it is observed here that due to holes in the ground plane of the perforated antenna, less than 1 dB reduction in the gain is seen compared to the gain of the Koln-based non-perforated RRMPA at the 2.45 GHz resonance frequency.

As seen in all the radiation graphs, the insertion of holes in the conductive layers of the perforated RRMPAs was the reason for the reduction in their realized gains. The least reduction in the realized gain was found for the perforated Koln-based RRMPA when compared to the other two perforated RRMPAs. For wearable applications requiring moderate antenna gains, the Koln-based non-perforated RRMPA can be a suitable option as it achieved moderate realized gain with not much difference at both the E-plane and the H-plane. Table 6 tabulates the realized gains of all the non-perforated and perforated RRMPAs.

### 4.4. Water Vapor Transmission Rates—WVTR of Antennas

The WVTR of the Koln-based non-perforated RRMPA was found to be 4409.44 g/m^2^/day, which is higher compared to the WVTR test results of the rest of the non-perforated RRMPAs. This is because Koln electrotextile offers higher AP at lower thickness compared to other electrotextiles used in this research work. In addition, when perforations of 1 mm diameter were added in the conductive layers of Koln-based perforated RRMPA, it helped to achieve the WVTR of 5296.70 g/m^2^/day. In contrast, Prag-based non-perforated RRMPA showed the lowest WVTR of 3707.42 g/m^2^/day. This is because of the compact woven structure of Shieldex^®^ Prag electrotextile, which offers low AP. The insertion of perforations of 1 mm diameter in Prag-based perforated RRMPA helped to improve its WVTR but still offers the lowest WVTR among other perforated RRMPAs. Therefore, for wearable applications at high relative humidity and temperature (38 °C ± 1° and 90% ± 2%, respectively), the Koln-based perforated RRMPA can be the most suitable breathable antenna that can help water vapors dissipate into the environment slightly more quickly compared to other antennas employed in this paper. Adding a large number of small-sized holes in the conductive layers of the antenna helped in achieving more AP and WVTR; hence, this novel technique to make a textile antenna breathable works efficiently. Table 7 tabulates the WVTR results from all non-perforated and perforated antennas. To walk at the rate of 5 km/h, the wearable fabric must allow moisture between 2800 and 7000 g/m^2^ per 24 h [31] for the wearer to feel and remain comfortable. All the non-perforated and perforated antennas employed in this research work fall within this range. Therefore for wearable applications, a wearer during physical activity such as walking can still feel comfortable wearing the antennas employed in this research work. During physical activity, perspiration helps to lose body heat when body temperature starts to rise and the fabric needs to allow perspiration in its vaporous form to pass through it at an elevated rate. As can be seen in Table 7, the perforations increase the WVTR with 221 g/m^2^/day (+5%) for the Kiel-based, a limited improvement, but up to 887 g/m^2^/day (+17%) for the Koln-based. The Koln-based perforated antenna if embedded in another wearable breathable garment will not affect the breathability of the garment as it is the most breathable antenna employed and will therefore be a suitable antenna for wearable applications.

## 5. Conclusions

Breathable textile RRMPAs for wearable applications were successfully designed and implemented. Enhanced breathability was observed when a large number of small-sized holes of 1 mm diameter in the conductive fabrics of RRMPAs were inserted. The simulation tool used in previous research was expanded by adding the perforations. Validation has shown the software tool can predict the behavior of the perforated antenna with sufficient accuracy. Simulations showed adding perforations did not impede the antenna which remained effective at a resonance frequency of 2.45 GHz (ISM band). Experiments confirmed this conclusion for all three different conductive fabrics tested. Every constructed and measured breathable textile RRMPA succeeded in combining enhanced breathability with sufficient antenna gain.

Out of all antenna prototypes studied, the Koln-based breathable perforated antenna prototype showed the most promising results, with a WVTR of 5296.70 g/m^2^/day, and a realized gain of 4.2 dBi and 5.4 dBi at E-plane and H-plane, respectively. In contrast, the Prag-based non-perforated RRMPA was the least air-permeable antenna, with a WVTR of 3707.42 g/m^2^/day of WVTR, and gains of 5.8 dBi and 3.3 dBi in the E-plane and H-plane, respectively. Thanks to the enhanced breathability of the proposed textile antenna, the effects of wetting caused by for instance sweating are minimized to a great extent as the humidity is transported much faster than in the standard textile antennas.

The antennas developed in this research consisted of a 3D knitted hydrophobic spacer fabric as an antenna substrate combined with laminated copper-coated polyamide electro textiles in which a regular pattern of small holes was cut. This novel approach successfully enables the creation of breathable textile antennas that offer all properties needed for textile use, flexibility, breathability and robustness, without losing the required antenna gain for use in the ISM band. The antenna leads to enhanced wear comfort sensation. It covers the entire 2.45 GHz ISM band with sufficient extra margin to allow for some tolerances in the production.

## Figures and Tables

**Table 1:** Properties of the conductive materials

* Data Given by the Manufacturer	Shieldex^®^ Prag	Shieldex^®^ Kiel-SK-96	Shieldex^®^ Koln
Fabric Type *	Woven	Nonwoven	Nonwoven
Surface resistivity * (Ω/m^2^)	<0.05	<0.02	<0.06
Thickness * (mm)	0.08 ± 10%	0.46 ± 10%	0.16 ± 10%
Weight * (g/m^2^)	82 ± 10%	104 ± 10%	80 ± 10%
Average air permeability (mm/s)	41.2 ± 2%	751 ± 2%	868 ± 2%

**Table 2:** Properties of the 3D perforated dielectric substrate.

	Thickness (mm)	Dielectric Constant (Ɛ_r_)	Tan δ	Average Air Permeability (mm/s)
3D Perforated Substrate	3 ± 10%	1.17	0.002	1545 ± 2%

**Table 3:** Dimensions of non-perforated and perforated textile RRMPAs.

Parameters	Abbreviation	Dimension (mm)
Length of patch	Lp	49
Width of patch	Wp	51
Length of ring	Lr	10
Width of ring	Wr	9
The diameter of holes (perforated antenna)	Ø_h_	1
Position of the feed	(*x*-axis, *y*-axis)	(6, 8)

**Table 4:** The air permeability of non-perforated and perforated RRMPAs.

		Shieldex^®^ Prag	Shieldex^®^ Kiel-SK-96	Shieldex^®^ Koln
Air Permeability (mm/s)	Non-perforated antenna	158 ± 2%	195 ± 2%	400 ± 2%
	Perforated antenna	322 ± 2%	389 ± 2%	510 ± 2%

**Table 5:** Variance analysis results of air permeability tests for non-perforated and perforated antennas.

	*F*-Value	*p*-Value
Group A	510.40	0
Group B	7885.79	0

**Table 6:** Measured realized gains of non-perforated and perforated RRMPAs.

		Shieldex^®^ Prag		Shieldex^®^ Kiel-SK-96		Shieldex^®^ Koln	
		E-Plane	H-Plane	E-Plane	H-Plane	E-Plane	H-Plane
Realized Gain (dBi)	Non-perforated antenna	5.8	3.3	7.3	4.0	4.9	5.9
	Perforated antenna	5.3	2.0	3.6	1.9	4.2	5.4

**Table 7:** Water Vapor Transmission Rate of all non-perforated and perforated antennas.

S.No	Antenna Prototypes	WVTR (g/m^2^/day) of Non-Perforated RRMPAs	WVTR (g/m^2^/day) of Perforated RRMPAs
1	Shieldex^®^ Prag	3707.42	4103.69
2	Shieldex^®^ Kiel-SK-96	4378.41	4599.65
3	Shieldex^®^ Koln	4409.44	5296.70

## Data Availability

All the relevant data presented in this study is contained within this article.
